# Phases of Professional Development

**Published:** 1876-08

**Authors:** W. C. Horne

**Affiliations:** Rome.


### ARTICLE V.

### *Phases of Professional Development.*

BY W. C. HORNE, D. D. S., ROME.

The rise of dentistry in the United States, as a recognized profession, is recent enough to be within the memory of practitioners still living: its growth has been rapid, its development in keeping with the progressive spirit of the country. Its early advancement was largely due to a few individuals, whose assiduity led them to surpass in their operations the very great number who, as in other callings, are content to follow afar off. The time has not arrived when the roll of honor of all these pioneers may be called. Death has removed many of them, and the ranks which have begun to be thinned must show ere long still many a gap.

Thirty or more years ago New York had attracted by her metropolitan fame and wealth a number of the foremost dentists of the country, of whom some still linger. Philadelphia, Boston and Baltimore each had its celebrities while scattered throughout the land were others, the luster of whose abilities shed a greater or lesser halo about them. It is to the labors of such, together with their compeers in Europe (then few in number,) that American dentistry owes the beginning of the high consideration which it enjoys at home and abroad. Let us pay the grateful tribute which is their due, content if our works shall also merit the praise of those who come after us.

Dr. Eleazer Parmly lived to see, in his own day, an advancement in the profession of which he was an ornament, such as falls to the lot of few men to witness, leaving an example of the gentler virtues which so endear the professional man to his clients. His career, almost coeval with that of our profession, embraces an epoch of great and varied interest, of which the souvenirs should be carefully gathered and piously preserved. Let us embalm his memory with the spices of his virtues, while with tender hands we draw the veil upon his frailties, with that charity of which we shall all at the last stand greatly in need.

Another of the blessed departed who came in and went out as a father before us, with the light of a true heart beaming in his face, was Dr. George E. Hawes. Gifted with a clear perception of the subjects which occupied the thoughts, and with a full comprehension of the objects which incited the efforts of dental practitioners, with a ready and kindly wit he resolved many a contested point, throwing light upon what was doubtful, and effectually disposing of crudities and absurdities, and that with apt illustration and agreeable humor. How much he was beloved and respected, with what satisfaction and confidence his advice and his decisions were accepted, the interest he took in every movement for the welfare of our profession, I need not detail to those who were the witnesses. He was one of the accomplished men of the old *regeme*, of whom some still remain to link our present with their brilliant past.

The labors of that noble man, Chapin A. Harris, whose name should be all the more honored for that he devoted his time and his talents less to the accumulation of wealth than to the development of dentistry into a scientific profession, together with his associates, lifted the practice of dentistry from that obscure empiricism which had largely characterized it into the clear light of scientific inquiry and demonstration, by laying that foundation in the Baltimore

Dental College upon which our system of dental education has been built.

So long as the various processes of the dental surgery and laboratory continue to be held as carefully-guarded secrets which might be filched from one by his neighbor, engendering a narrow-minded jealousy of every means and appliance of which one had not the monopoly, so long there could be neither professional character nor standard; and to the disciple of such a school it was enough to be as his master. But the leaven of the new order of things began to work. The aspiration for better instruction, necessary to the attainment of a professional standing, began to imbue the minds of younger practitioners, encouraged by the success of their seniors and incited by the desire of popular approval. The granting of honorary diplomas was doubtless a means of giving popularity to the new enterprise, and was intended to create a professional *esprit du corps.* The occasion, or the supposed necessity for this policy, has long since passed away; the advantages for instruction in the branches of dental art and science are now so widespread, that a thorough and increasingly expansive course of study should be demanded from the neophytes who henceforth present themselves.

In pursuing the inquiry which frequently recurs, "To what is due the superiority of American Dentists?" acknowledgment must be made of the fertility of the American mind in originating appliances for the perfection of given operations, or combining or improving upon those already known; of the avidity with which new and intricate processes producing approved results are siezed on and turned to immediate use by those interested, under the spur of a constant public demand for what is newest and best; and of the freedom of inquiry (running at times far into the realms of speculation) which has characterized some of our best men, added to their liberality in giving freely to their fellows the fruit of months or years of experiment and investigation, and the diffusion, with an almost apostolic spirit, of the light which has shone upon them. A large meed of praise is due to our dental colleges whose professors have devoted much time and pains to the instructions of those who have sat at their feet. But the instruction afforded by the dental college is necessarily of a preliminary character; the diploma granted in the usual order certifies that the bearer has studied and been examined in a certain curriculum, and is qualified to practice. But excellence in his profession is to be gained by years of intelligent observation and experience, while eminence requires, in addition, a rare combination of faculties and acquirements. While our dental schools are, then, the proper entrance into the course of life designated by the term "Dentist," there is necessary a higher school for the attainment of a larger and more varied knowledge.

The Dental Society is the arena where earnest and studious men may present their theories and their discoveries for discussion and for demonstration: where that which is new and good is sure to find recognition and praise; where what is wrong, bad, or imperfect, will be detected and exposed, and where worth and presumption will each very soon meet its proper distinction and reward. One who is a Sir Oracle to the circle of his local admirers, and comes in all the glory of delegateship to the annual reunion at Niagara, or Saratoga, or elsewhere, is soon toned down into a wholesome perception of the smallness of the horizon which his eye has been accustomed to scan, and finds that there are more things in heaven and earth than were dreamed of in his philosophy. He goes home a wiser and a better man; and his brethren of the Mad River Valley, or the Thunder and Lightning Hills, learn, in their turn, that there are other worlds than ours.

To ignore or contemn the visions of an original mind is worse than a blunder. The world is indebted for much of its advancement to its dreamers, who supply the ideals which another order of intelligence works out. Horace Wells demonstrated the practicability of anæsthesia with the protoxide of nitrogen; it remained for others to elaborate the appliances which make it generally available. What seer will call down upon his head the blessings of the race by the discovery and demonstration of a reliable process of inducing the exposed dental pulp to recover itself, or to accept kindly the juxtaposition of some foreign substance which shall not excite its exquisite sensibilities?

It has been due to the demand found to be general, to fragmentary ideas gathered here and there, to the incentive of praise bestowed and rewards gained, that many of our most useful appliances have been engendered and brought to light through the media of dental societies, affording as they do admirable opportunities for keeping *au courant* with the progress or ideas of the day. It it no argument against the value of such associations that they have sometimes been scenes of dispute or disorder; that they may have been due to the absence of a clear head and a controlling hand at the helm. Such accidents, unpleasant and pernicious, are capable of being avoided or guarded against. A democratic disdain of rules and regulations has often been seen very seriously to interfere with the decorum and repose necessary to a calm reception and investigation of subjects, whether purely scientific or eminently practical. The declamation of the stump orator, and the wire-pulling of the politician, are not the proper adjuncts of a scientific society, and the natural sequence of their toleration is loss of prestige with those who reprobate such proceedings. This has been fully evinced in the history of some of our dental societies.—*Dental Cosmos.*

